# Determination of Antimicrobial Activity of Extracts of Indigenous Wild Mushrooms against Pathogenic Organisms

**DOI:** 10.1155/2019/6212673

**Published:** 2019-02-18

**Authors:** Gebreselema Gebreyohannes, Andrew Nyerere, Christine Bii, Desta Berhe Sbhatu

**Affiliations:** ^1^Department of Biological and Chemical Engineering, Mekelle Institute of Technology, Mekelle University, Ethiopia; ^2^Molecular Biology and Biotechnology, Pan African University, Institute for Basic Sciences, Technology, and Innovation; Jomo Kenyatta University of Agriculture and Technology, Nairobi, Kenya; ^3^Department of Medical Microbiology, College of Health Sciences, Jomo Kenyatta University of Agriculture and Technology, Nairobi, Kenya; ^4^Center for Microbiology Research, Kenya Medical Research Institute, Nairobi, Kenya

## Abstract

**Objective:**

This study has investigated the antimicrobial activity of extracts of indigenous wild mushrooms against selected organisms.

**Methods:**

Thirty-five (35) indigenous wild mushrooms were collected from Arabuko-Sokoke and Kakamega National Reserve Forests, Kenya. All mushrooms were identified and their contents were extracted and screened for their antimicrobial activities against* Escherichia coli *(clinical isolate)*, Klebsiella pneumoniae* (ATCC 13883),* Pseudomonas aeruginosa* (clinical isolate),* Pseudomonas aeruginosa* (ATCC 27853),* Staphylococcus aureus *(ATCC 25923), MRSA (ATCC 33591),* Candida albicans* (clinical isolate), and* Candida parapsilosis* (ATCC 90018) using tetrazolium microtiter plate bioassay method.

**Results:**

Of the 35 tested mushroom extracts, extracts of three (3) mushrooms, namely,* Trametes *spp. (Arabuko-Sokoke forest),* Trametes*, and* Microporus* spp. (Kakamega forest), have shown promising antimicrobial activities against the tested organisms. The* S. aureus* (ATCC 25923),* P. aeruginosa* (ATCC 27853), and Methicillin-Resistant* Staphylococcus aureus* (MRSA) (ATCC 33591) were the most susceptible to chloroform extract of* Trametes* spp. collected from Arabuko-Sokoke forest. Of the tested organisms,* S. aureus* (ATCC 25923) was the most susceptible whereas* E. coli* was the most resistant organism to the hot water extract of* Trametes* spp. collected from Arabuko-Sokoke forest. Chloroform extract of* Microporus *spp. has shown the highest antibacterial activity against* S. aureus* (ATCC 25923), MRSA (ATCC 33591), and* K. pneumoniae* (ATCC 13883) but limited activity against* E.coli*. All extracts of the three wild mushrooms have shown the most antibacterial activities against* S. aureus *(ATCC 25923).

**Conclusion:**

The present study has shown that the extracts of the three wild mushrooms have shown promising antimicrobial activities against the tested organisms.

## 1. Introduction

Nowadays, the world is facing significant challenges in modern healthcare services because many antimicrobial agents have lost their effectiveness in treating infectious diseases primarily due to the development of microbial resistance [1]. Exploration for bioactive compounds effective in treating pathogenic microorganisms resistant to present-day drugs is very helpful [2]. Currently, there is a growing interest in searching for new antimicrobial agents from natural sources such as bacteria, fungi, and plants [3, 4]. Natural products, especially microbial and plant products, constitute the major sources of new drug molecules [1].

Mushroom species release various bioactive compounds such as terpenoids, flavonoids, tannins, alkaloids, and polysaccharides [5, 6]. Mushrooms are immensely rich in bioactive compounds yet largely untapped resource of useful natural compounds. These bioactive compounds are found in various cellular components and secondary metabolites, which have been isolated and identified from the fruiting bodies [6]. The fruiting bodies and mycelium of mushrooms exhibit health promoting values such as immunostimulatory, antibacterial, and antioxidative properties [7]. The synergistic effect of these substances would give potential therapeutic values.

## 2. Materials and Methods

### 2.1. Collection and Identification of Mushrooms and Extraction of Their Contents

The wild mushrooms were collected from Arabuko-Sokoke and Kakamega National Reserve forests in Kenya. They were identified by comparing their morphological characters against related literature [5]. Mushroom extraction was carried out using chloroform, 70% ethanol, and hot water solvents [6, 7]. A 100 g of powdered mushroom was mixed with 0.5 L of each solvent in a conical flask at 25°C and was shaken using an incubator shaker at 150 rpm for 72 h. The extracts were centrifuged at 3000 rpm for 15 min, filtered with Whatman No.1 filter paper, and evaporated and dried using a rotary evaporator at 50°C. The extracts were kept in -80°C deep freezer and freeze-dried by a freeze-dryer. Finally, they were stored in the 4°C refrigerator in an amber colored bottle for further analyses.

### 2.2. Preparation of Test Organisms

Six bacterial species, namely,* Escherichia coli *(clinical isolate)*, Klebsiella pneumoniae* (ATCC 13883),* Pseudomonas aeruginosa* (clinical isolate),* Pseudomonas aeruginosa* (ATCC 27853),* Staphylococcus aureus *(ATCC 25923), and Methicillin-Resistant* Staphylococcus aureus* (MRSA) (ATCC 33591), as well as two yeast species, namely,* Candida albicans* (clinical isolate) and* Candida parapsilosis* (ATCC 90018), were selected as test organisms. Whereas bacterial test organisms were grown in 5 mL of Mueller-Hinton broth at 37°C for 12–16 h, the yeast test organisms were grown in 5 mL Sabouraud dextrose broth at 30°C for 24 h. The inoculum size of each test organism was adjusted to a concentration of 1.5×10^8^ CFU/mL by comparing with 0.5 McFarland standards.

### 2.3. Determination of Antimicrobial Activities of Extracts of Wild Mushrooms

The antimicrobial activities of extracts of wild mushrooms were determined by establishing the minimum inhibitory concentration (MIC) of the extracts using tetrazolium microtiter plate bioassay method [8–11]. For this purpose, stock solutions of mushroom extracts and Mueller-Hinton broth were prepared. The stock solution of each mushroom extract was prepared by dissolving 20 mg/mL of extract in dimethyl sulfoxide (DMSO).

First, 100 *μ*L of Mueller-Hinton broth was poured into each of the 96 (8 rows by 12 columns) wells of the microtiter plate. Then, the 100 *μ*L mushroom extract was added to Well #1 in Row A containing the broth, and the contents were mixed and diluted in serial twofold dilutions through Well #9 yielding concentrations ranging from 2 mg/mL (for Well #1) to 7.813 *μ*g/mL (for Well #9). The 100 *μ*L content supposed to be transferred to Well #10 was discarded; thus no extract was added afterward. Thus, Wells #10, #11, and #12 were left with no mushroom extract and designated as “positive control”, “quality control”, and “negative control”, respectively. Wells #1 through #9 in the remaining rows (rows B through H) were filled with a mixture of Mueller-Hinton broth and mushroom extracts with similar concentrations, leaving the last three wells for positive, quality, and negative controls.

With the exception, Well #11 in each row, the contents of Wells #1 through #12 were inoculated with 100 *μ*L of test/target organism in triplicate. Chloramphenicol and Clotrimazole drugs were dissolved in a sterile distilled water to a final concentration of 0.1 *μ*g/mL to serve as a positive control against bacterial and yeast species, respectively. The microtiter plate was sealed with parafilm and incubated at 37°C and 30°C for 24 h for bacterial and yeast species, respectively. After 24 h of incubation, the MIC of each test organism was detected by adding 50 *μ*L of 0.2 mg/mL indicator dye of 2, 3, 5-triphenyltetrazolium chloride (TTC) into the wells of the microtiter plate. Then, the microtiter plate was incubated for 30 min to 3 h at 37°C and 30°C for bacterial and yeast species, respectively. Any color change of the TTC was checked every 30 min for 3 h. Biologically active bacterial and yeast cells reduce the TTC to colorless TTC salt and pinkish-red formazan product. When solutions in the wells of microtiter plate remained clear (without color change), we infer that the bacterial and yeast cells were inhibited. The minimum bactericidal concentration (MBC) and minimum fungicidal concentration (MFC) of the contents were determined by taking a loopful of inoculum from each well of the microtiter plate with clear content (without any color change) and streaked it on Mueller-Hinton and Sabouraud dextrose agar plates for bacterial and yeast species, respectively. The bacteria and yeast-streaked plates were incubated for 24 h at 37°C and 30°C, respectively. Then, the MBCs and MFCs were determined as the lowest concentration of the extract that permit no growth of bacteria and yeast, respectively.

### 2.4. Statistical Analysis

All quantitative data were compared using relevant descriptive and inferential statistics at* a priori* significance level of* p* ≤ 0.05.

## 3. Results

Of the 35 tested wild mushrooms, three (3) of them, namely,* Trametes *spp. (Arabuko-Sokoke forest),* Trametes *spp., and* Microporus* spp. (Kakamega forest), have shown promising antimicrobial activities against six bacterial pathogens, namely,* Staphylococcus aureus *(ATCC 25923), Methicillin-Resistant* Staphylococcus aureus* (MRSA) (ATCC 33591),* Klebsiella pneumoniae* (ATCC 13883),* Pseudomonas aeruginosa* (ATCC 27853),* Pseudomonas aeruginosa* (clinical isolate), and* Escherichia coli* (clinical isolate), as well as two yeast species called* Candida albicans* (clinical isolate) and* Candida parapsilosis* (ATCC 90018).

### 3.1. Trametes Extracts

The antimicrobial activities of chloroform, 70% ethanol, and hot water extracts of* Trametes* spp. were quantitatively analyzed against eight clinical isolates and standard strains (Table 1). Among the tested human pathogens,* S. aureus* (ATCC 25923),* P. aeruginosa* (ATCC 27853), and MRSA (ATCC 33591) were the most susceptible species to chloroform extract with MIC values of 0.83±0.29 mg/mL, 1.00 mg/mL, and 1.17±0.76 mg/mL, respectively. Chloroform extracts have resulted in a moderate growth inhibition against* C. albicans* and* C. parapsilosis* (ATCC 90018) at MIC value of 1.50±0.87 mg/mL. On the other hand,* S. aureus* (ATCC 25923) and MRSA (ATCC 33591) were the most susceptible to 70% ethanolic extract at MIC values of 0.67±0.29 mg/mL and 0.83±0.29 mg/mL, respectively. Though all the tested organisms were inhibited by the hot water extract, the* S. aureus* (ATCC 25923) was the most inhibited Gram-positive bacterium. Moreover, chloroform and ethanol (70%) extracts have resulted in good growth inhibitory activities against the* S. aureus* (ATCC 25923).

Compared to the positive control, all the three extracts have resulted in some antimicrobial activity against all the tested organisms. Hot water extract has resulted in the most powerful antimicrobial activity against all tested organisms. The antimicrobial activities of the three extracts against the tested organisms were calculated and compared. But statistically significantly different antimicrobial activities were observed only between the hot water and chloroform extracts against* E. coli *(*df* = 2, F = 7.00,* p *≤ 0.024). Hot water extracts have shown the strongest antimicrobial activities against all the tested organisms (Table 1). Chloroform extracts have shown statistically significantly different antimicrobial activities against* S. aureus *(ATCC 25923),* P. aeruginosa* (clinical isolate),* C. albicans *(clinical isolate), and* C. parapsilosis* (ATCC 90018) (*df* = 7, F = 3.81,* p *≤ 0.05).

### 3.2. Trametes Extracts

Extracts of* Trametes* spp. have exhibited different degrees of antimicrobial activities against the tested organisms (Table 2). Even though* C. albicans* and* C. parapsilosis* (ATCC 90018) were found to be susceptible to chloroform, ethanol, and hot water extracts, they were resistant to positive control (clotrimazole).* S. aureus* (ATCC 25923) was the most susceptible bacterium to hot water extract with a MIC value of 0.50 mg/mL.

**Figure 1:**
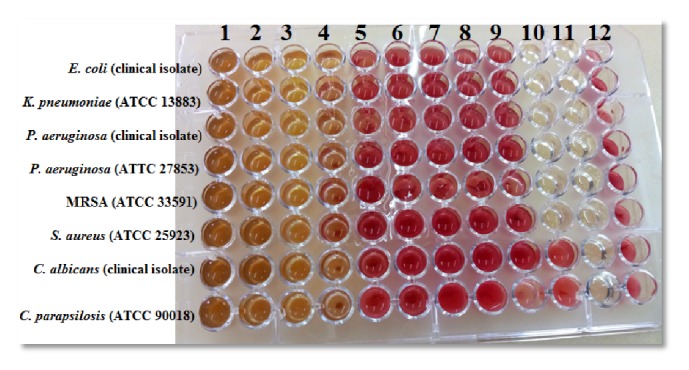
Determination of minimum inhibitory concentrations of the chloroform extract of* Trametes* spp. against clinical isolates and standard strains. The numbers from 1 to 9 at the top of the microtiter plate indicate the different concentrations of the extract in the wells, i.e., well-1(2mg/mL); well-2 (1mg/mL); well-3 (0.5mg/mL), well-4 (0.25mg/mL); well-5 (0.125mg/mL); well-6 (0.62*μ*g/mL); well-7 (0.031*μ*g/mL); well-8 (0.015 *μ*g/mL); well-9 (0.0078*μ*g/mL); well-10-positive control; well-11-quality control; well-12-negative control.

The chloroform extract of* Trametes* spp. has resulted in varied antimicrobial activities against* E. coli*,* P. aeruginosa* (ATCC 27853), and MRSA (ATCC 33591) (Figure 1). Except for* P. aeruginosa* (clinical isolate), ethanol extracts of* Trametes* spp. have shown stronger antimicrobial activities against the bacterial species compared to the fungal species (Table 2). The Gram-negative bacterium,* S. aureus* (ATCC 25923), was the most susceptible one to hot water extract with MBC value of 0.5 mg/mL. Chloroform, ethanol, and hot water extracts were compared for their antimicrobial activities against* S. aureus* (ATCC 25923). But only chloroform and water extracts have shown statistically significantly different antibacterial activity against the bacterium (*df* = 2, F = 25.29,* p *≤ 0.05).

### 3.3. Microporus Extracts

Extracts of* Microporus* spp. were tested for their microbial activities. Chloroform extracts of this group have resulted in higher antibacterial activities against* S. aureus* (ATCC 25923), MRSA (ATCC 33591), and* K. pneumoniae* (ATCC 13883) as compared to* E. coli* (Table 3). All extracts have resulted in good growth inhibitory activities against* S. aureus* (ATCC 25923) and MRSA (ATCC 33591). However, ethanol and hot water extracts have resulted in the limited effect on clinical isolates of* E. coli* and* P. aeruginosa*. Statistically significant different mean growth inhibitory activities were observed between chloroform and hot water extracts of* Microporus* spp. against* E. coli *(*df* = 2, F = 25.29,* p *≤ 0.05). The extracts have also shown marked differences in their antimicrobial activities between the clinical isolates and standard strains of the tested organisms (Table 3). The clinical bacterial and yeast isolates were found to be more resistant to the extracts compared to the standard strains. Comparatively speaking, chloroform, ethanol, and hot water extracts have resulted in better antimicrobial activities against* S. aureus* (ATCC 25923). The antimicrobial activities of chloroform and hot water extracts against* E. coli* were statistically significantly different (*df* = 2, F = 25.29,* p *≤ 0.05). Likewise, the antibacterial activities of chloroform and hot water extracts against a clinical isolate of* P. aeruginosa* were statistically significantly different (*df* = 2, F = 25.29,* p *≤ 0.05).

## 4. Discussion

Currently, incidences of multidrug-resistant organisms are increasing and compromising the treatment of a growing number of infectious diseases. As a result, there is an urgent need for the development of new and effective drugs against current antibiotic-resistant pathogens [12]. Fungal species have been proven to be outstanding potential sources of bioactive compounds of high therapeutic value. They are also the richest sources of secondary metabolites [13]. The three mushrooms genera addressed in this article have shown promising antimicrobial activities against the tested organisms. Ethanol extracts of* Trametes* spp. have resulted in excellent antibacterial activities against* S. aureus* (ATCC 25923) and MRSA (ATCC 33591), human pathogenic bacteria. These bacteria were the most susceptible to ethanol extract. In general, chloroform, ethanol, and hot water extracts of* Trametes* spp. have shown the highest antibacterial activity against* S. aureus* (ATCC 25923).

In regard to the choice of the solvent of extraction, hot water extraction has yielded good extracts with better antimicrobial activities against all of the tested organisms. Since water is more polar solvent compared to chloroform and ethanol, it easily penetrates into the intracellular matrix of the mushrooms cell wall [14]. Unlike the less polar solvents, more polar solvents are more effective in extracting organic and inorganic compounds [8]. These findings were supported by results of a study by Davi and Krishnakumari [9] who have reported the existence of a high level of flavonoid in hot water extracts. The increased polarity of hot water leads to increased extraction of bioactive compounds (such as alkaloids, saponins, tannins, flavonoids, and terpenoids) leading to increased antimicrobial activities against the tested organisms [10]. In this case too, our results are similar to findings of a previous study done on the antimicrobial activities of some local mushrooms on human pathogenic isolates. Ethanol and hot water extracts of the mushrooms contained higher bioactive substances than cold water extracts [11].

Unlike chloroform and ethanol extracts, hot water extracts have resulted in the strongest antimicrobial activity against all tested organisms. The hot water extraction might produce many antimicrobial compounds such as flavonoids, tannins, and terpenoids. This observation agrees with the findings of a study on the antimicrobial activities of whole fruiting bodies of* Trametes hirsute* against some common pathogenic bacterial and fungal species. The study has found out that water extracts were better than methanol extracts against bacterial and fungal pathogens [15]. Another study has also reported that antimicrobial activities of wild mushroom extracts obtained from different solvents vary [16]. Likewise, water extracts of wild mushrooms were found to be more effective against* P. aeruginosa*,* E. coli*,* B. subtilis*, and* C. albicans* isolates [17] in support of our findings. Interestingly, a study on the evaluation of the antimicrobial activities of chloroform extracts of* Trametes versicolor* against* S. aureus* and* E. faecalis* was found to be profound. The reason for this unexpectedly higher antimicrobial activities of* Trametes versicolor* against* S. aureus* and* E. faecalis* is argued to be due to differences in the geographical locations of the habitats of the wild mushrooms [18].

The lower antimicrobial activities of chloroform extracts can be explained by the absence of potent secondary metabolites due to the effectiveness of the extraction method, the extraction capacity of the solvent, any differences in the solubilities of the various bioactive compounds, the extraction time, etc. [19]. Chloroform extracts of fruiting bodies of* Trametes* spp. resulted in weak inhibitory effects towards the target/tested organisms. This could be due to the fact that many antimicrobial compounds such as flavonoids and terpenoids are polar and they cannot be extracted using the less polar solvent of chloroform. Thus, chloroform extracts appeared to be the least active compared to extracts by other polar solvents [8]. The results of our study with chloroform, ethanol (70%), and hot water solvents indicate that the bactericidal and fungicidal activities of the extracts increase with increasing the polarity of the solvents. This observation was supported by one study that has evaluated the antibacterial activities of Australian basidiomycetous macrofungi using a high-throughput 96-well plate assay [20]. Another study with water extract of* Agaricus* spp. has resulted in good antimicrobial activity against both Gram-positive and Gram-negative bacterial species [4]. Generally speaking, antimicrobial activities of chloroform, ethanol (70%), and hot water extracts among target/tested organisms differ with the polarity of the solvents. A deviation from this trend, however, is argued to be due to differences in the origins of the mushrooms used and/or the nature of bacterial strains targeted/tested [21].

Mushroom extracts have shown varied antimicrobial activities against Gram-positive and Gram-negative bacteria. This observation agrees with findings of a study on antimicrobial activities and mineral compositions of shiitake mushrooms cultivated on agricultural wastes [22]. In the case of the present study,* S. aureus *(ATCC 25923), a Gram-positive bacterium, is found to be highly susceptible and inhibited to chloroform, ethanol (70%), and hot water extracts compared to other tested/targeted organisms. This is supported by a previous study where chloroform and water extracts of wild mushrooms have good antibacterial activities against* S. aureus. *Hot water extracts have also shown antibacterial activities against the tested/targeted organisms and the results were comparable with the antibacterial activities of ampicillin [22].

On the other hand, Gram-positive bacteria such as* E. coli*,* K. pneumoniae* (ATCC 13883),* P. aeruginosa* (clinical isolate), and* P. aeruginosa* (ATTC 27853) were the most resistant to almost all extracts. These results clearly confirm that Gram-positive bacteria are highly susceptible to the extracts and were in agreement with the previous reports [11, 23]. For example, Akyuz and Kirbag [24] concluded that the extracts of* Agaricus bisporus* and* Pleurotus florida* were found to have better inhibitory activities against Gram-negative compared to Gram-positive bacteria. Other reports have also suggested that the difference in susceptibility to antimicrobial extracts between Gram-positive and Gram-negative bacteria could be attributed to morphological differences. For example, the outer membrane of Gram-negative bacteria has lipopolysaccharide, making the cell wall impermeable to lipophilic extracts [14, 25].

Extracts of* Trametes* spp. used in this study have exhibited varying degrees of antimicrobial activities within the same Gram reaction organisms. The observed variation in the antimicrobial activities of the extracts in the same Gram reaction organisms might be linked to their ability in producing capsule and slime layers as well as the presence of resistance factors like plasmids, transposons, and insertion sequences. Previous studies also suggested that the differences in antimicrobial activities might be due to a number of factors, including the genetic makeups of the test organisms, the solvents used in the extraction of bioactive agents, and the differences in the physical and biochemical nature of the antimicrobial components of the extracts [8, 26].

All extracts of the present study have shown a marked difference in their antimicrobial activities towards the clinical isolates and standard strains. The clinical bacterial and yeast isolates were found to be more resistant to the extracts than the standard strains. The main reason for the resistance of the clinical isolates might be directly linked to the indiscriminate exposure of the clinical isolates to various antimicrobial agents. According to the Taiwo [27], several clinical isolates have effective antibiotic resistance mechanisms through the acquisition of resistant genes, production of enzymes (e.g., ß-lactamases), and efflux pumping of the drug out of the cell.

In conclusion, all extracts of the three wild mushrooms have shown potent antimicrobial activities against the tested/targeted organisms. Further investigation is needed to evaluate and confirm the antimicrobial activities of the extracts against a wider range of human pathogenic microorganisms. Finally, isolation, identification, and explanation of the mode of action of the bioactive compounds responsible for the antimicrobial activities are indispensable steps prior to the development of antibiotics.

## Figures and Tables

**Table 1:** Antimicrobial activities of crude extracts of *Trametes* spp. collected from Arabuko-Sokoke forest against clinical isolates and standard strains.

Tested organisms	Chloroform extract (mg/mL)			70% ethanol extract (mg/mL)			Hot water extract (mg/mL)		
	MIC	MBC/MFC	+ve (*µ*g/mL)	MIC	MBC/MFC	+ve (*µ*g/mL)	MIC	MBC/MFC	+ve (*µ*g/mL)
*E. coli* (clinical isolate)	1.33±0.58^a^	1.67±0.58^a^	0.10±0.00^kl^	1.00±0.00^b^	1.33±0.58^kl^	0.10±0.00^kl^	0.83±0.29^kl^	1.17±0.76^kl^	0.10±0.00^kl^
*K. pneumoniae* (ATCC 13883)	1.33±0.58^kl^	1.67±0.58^kl^	0.10±0.00^kl^	1.00±0.00^kl^	1.33±0.58^kl^	0.10±0.00^kl^	0.83±0.29^kl^	0.83±0.29^kl^	0.10±0.00^kl^
*P. aeruginosa* (clinical isolate)	1.33±0.58^kl^	1.67±0.58^b^	0.10±0.00^kl^	1.33±0.58^kl^	1.00±0.00^kl^	0.10±0.00^kl^	1.00±0.00^kl^	1.00±0.00^kl^	0.10±0.00^kl^
*P. aeruginosa* (ATTC 27853)	1.00±0.00^kl^	1.33±0.58^kl^	0.10±0.00^kl^	1.00±0.00^kl^	1.33±0.58^kl^	0.10±0.00^kl^	0.83±0.29^kl^	0.83±0.29^kl^	0.10±0.00^kl^
MRSA (ATCC 33591)	1.17±0.76^kl^	1.00±0.00^kl^	0.10±0.00^kl^	0.83±0.29^kl^	1.00±0.00^kl^	0.10±0.00^kl^	0.83±0.29^kl^	1.00±0.00^kl^	0.10±0.00^kl^
*S. aureus* (ATCC 25923)	0.83±0.29^kl^	0.83±0.29^c^	0.10±0.00^kl^	0.67±0.29^kl^	0.83±0.29^kl^	0.10±0.00^kl^	0.67±0.29^kl^	0.67±0.29^kl^	0.10±0.00^kl^
*C. albicans* (clinical isolate)	1.50±0.87^kl^	1.67±0.58^d^	0.10±0.00kl	1.00±0.00^kl^	1.33±0.58^kl^	0.10±0.00^kl^	0.83±0.29^kl^	1.33±0.58^kl^	0.10±0.00^kl^
*C. parapsilosis* (ATCC 90018)	1.50±0.87^kl^	1.67±0.58^d^	0.10±0.00^kl^	1.17±0.76^kl^	1.33±0.58^kl^	0.10±0.00^kl^	0.83±0.29kl	1.00±0.00^kl^	0.10±0.00^kl^

MIC: minimum inhibitory concentrations; MBC/MFC: minimum bactericidal/fungicidal concentrations; +ve: positive control. Values are mean ± SD of three replicates. Values marked by different superscript letters within a column are statistically significantly different at *p *≤ 0.05. Values marked by different superscript letters within a row are statistically significantly different at *p *≤ 0.05.

**Table 2:** Antimicrobial activities of the crude extracts of *Trametes* spp. collected from Kakamega forest against clinical isolates and standard strains.

Tested organisms	Chloroform extract (mg/mL)			70% ethanol extract (mg/mL)			Water extract (mg/mL)		
	MIC	MBC/MFC	+ve (*µ*g/mL)	MIC	MBC/MFC	+ve (*µ*g/mL)	MIC	MBC/MFC	+ve (*µ*g/mL)
*E. coli* (clinical isolate)	1.33±0.58^cc^	1.67±0.58^cc^	0.10±0.00^cc^	1.33±0.58^cc^	1.00±0.00^cc^	0.10±0.00^cc^	0.83±0.29^cc^	0.83±0.29^cc^	0.10±0.00^cc^
*K. pneumoniae* (ATCC 13883)	1.00±0.00^cc^	1.00±0.00^cc^	0.10±0.00^cc^	1.00±0.00^cc^	1.00±0.00^cc^	0.10±0.00^cc^	0.67±0.29^cc^	1.33±0.58^cc^	0.10±0.00^cc^
*P. aeruginosa* (clinical isolate)	1.67±0.58^cc^	1.67±0.58^cc^	0.10±0.00^cc^	1.67±0.58^cc^	1.33±0.58^cc^	0.10±0.00^cc^	1.00±0.00^cc^	1.00±0.00^cc^	0.10±0.00^cc^
*P. aeruginosa* (ATTC 27853)	1.33±0.58^cc^	1.00±0.00^cc^	0.10±0.00^cc^	1.33±0.58^cc^	1.00±0.00^cc^	0.10±0.00^cc^	1.00±0.00^cc^	0.83±0.29^cc^	0.10±0.00^cc^
MRSA (ATCC 33591)	0.83±0.29^cc^	1.00±0.00^cc^	0.10±0.00^cc^	0.83±0.29^cc^	0.83±0.29^cc^	0.10±0.00^cc^	0.83±0.29^cc^	0.83±0.29^cc^	0.10±0.00^cc^
*S. aureus* (ATCC 25923)	0.67±0.29^cc^	1.00±0.00^a^	0.10±0.00^cc^	0.67±0.29^cc^	0.83±0.29^cc^	0.10±0.00^cc^	0.50±0.00^cc^	0.50±0.00^b^	0.10±0.00^cc^
*C. albicans* (clinical isolate)	1.00±0.00^cc^	1.67±0.58^cc^	>0.10±0.00^cc^	1.00±0.00^cc^	1.33±0.58^cc^	0.10±0.00^cc^	0.83±0.29^cc^	1.00±0.00^cc^	0.10±0.00^cc^
*C. parapsilosis* (ATCC 90018)	1.00±0.00^cc^	1.67±0.58^cc^	>0.10±0.00^cc^	1.00±0.00^cc^	1.33±0.58^cc^	0.10±0.00^cc^	0.67±0.29^cc^	1.00±0.00^cc^	0.10±0.00^cc^

MIC: minimum inhibitory concentrations; MBC/MFC: minimum bactericidal/fungicidal concentrations; +ve: positive control. Values are mean ± SD of three replicates. Values marked by different superscript letters within a column are statistically significantly different at *p *≤ 0.05. Values marked by different superscript letters within a row are statistically significantly different at *p *≤ 0.05.

**Table 3:** Antimicrobial activities of crude extracts of *Mircoporus* spp. collected from Kakamega forest against clinical isolates and standard strains.

Tested organisms	Chloroform extract (mg/mL)			70% ethanol extract (mg/mL)			Water extract (mg/mL)		
	MIC	MBC/MFC	+ve (*µ*g/mL)	MIC	MBC/MFC	+ve (*µ*g/mL)	MIC	MBC/MFC	+ve (*µ*g/mL)
*E. coli* (clinical isolate)	2.00±0.00^a^	2.00±0.00^bb^	0.10±0.00^bb^	1.67±0.58^bb^	1.67±0.58^bb^	0.10±0.00^bb^	1.67±0.58^b^	1.67±0.58^bb^	0.10±0.00^bb^
*K. pneumoniae* (ATCC 13883)	1.33±0.58^bb^	1.67±0.58^bb^	0.10±0.00^bb^	1.00±0.00^bb^	1.33±0.58^bb^	0.10±0.00^bb^	1.00±0.00^bb^	1.33±0.58^bb^	0.10±0.00^bb^
*P. aeruginosa* (clinical isolate)	2.00±0.00^bb^	2.00±0.00^c^	0.10±0.00^bb^	1.67±0.58^bb^	1.67±0.58^bb^	0.10±0.00^bb^	1.67±0.58^bb^	1.67±0.58^d^	0.10±0.00^bb^
*P. aeruginosa* (ATTC 27853)	1.67±0.58^bb^	1.33±0.58^bb^	0.10±0.00^bb^	1.33±0.58^bb^	1.00±0.00^bb^	0.10±0.00^bb^	1.33±0.58^bb^	1.00±0.00^bb^	0.10±0.00^bb^
MRSA (ATCC 33591)	1.00±0.00^bb^	1.33±0.58^bb^	0.10±0.00^bb^	1.00±0.00^bb^	1.00±0.00^bb^	0.10±0.00^bb^	1.00±0.00^bb^	1.00±0.00^bb^	0.10±0.00^bb^
*S. aureus* (ATCC 25923)	0.83±0.29^bb^	1.00±0.00^bb^	0.10±0.00^bb^	0.67±0.29^bb^	0.83±0.29^bb^	0.10±0.00^bb^	0.67±0.29^bb^	0.83±0.29^bb^	0.10±0.00^bb^
*C. albicans* (clinical isolate)	1.67±0.58^bb^	2.00±0.00^bb^	0.10±0.00^bb^	1.33±0.58^bb^	1.67±0.58^bb^	0.10±0.00^bb^	1.33±0.58^bb^	1.67±0.58^bb^	0.10±0.00^bb^
*C. parapsilosis* (ATCC 90018)	1.33±0.58^bb^	1.67±0.58^bb^	0.10±0.00^bb^	1.33±0.58^bb^	1.33±0.58^bb^	0.10±0.00^bb^	1.33±0.58^bb^	1.33±0.58^bb^	0.10±0.00^bb^

MIC: minimum inhibitory concentrations; MBC/MFC: minimum bactericidal/fungicidal concentrations; +ve: positive control. Values are mean ± SD of three replicates. Values marked by different superscript letters within a column are statistically significantly different at  *p *≤ 0.05. Values marked by different superscript letters within a row are statistically significantly different at  *p *≤ 0.05.

## Data Availability

The data used to support the findings of this study are available from the corresponding author upon request.
